# Metabolic Traits in Cutaneous Melanoma

**DOI:** 10.3389/fonc.2020.00851

**Published:** 2020-05-19

**Authors:** Monica Neagu

**Affiliations:** ^1^Immunology Department, “Victor Babes” National Institute of Pathology, Bucharest, Romania; ^2^Pathology Department, Colentina University Hospital, Bucharest, Romania; ^3^Faculty of Biology, Doctoral School, University of Bucharest, Bucharest, Romania

**Keywords:** melanoma, metabolism, immune cells, tumor microenvironment, therapy

## Abstract

Tumor microenvironment is a network of complex cellular and molecular systems where cells will gain specific phenotypes and specific functions that would drive tumorigenesis. In skin cancers, tumor microenvironment is characterized by tumor infiltrating immune cells that sustain immune suppression, mainly lymphocytes. Melanoma cellular heterogeneity can be described on genetic, proteomic, transcriptomic and metabolomic levels. Melanoma cells display a metabolic reprogramming triggered by both genetic alterations and adaptation to a microenvironment that lacks nutrients and oxygen supply. Tumor cells present clear metabolic adaptations and identifying deregulated glycolysis pathway could offer new therapy targets. Moreover, the immune cells (T lymphocytes, macrophages, NK cells, neutrophils and so on) that infiltrate melanoma tumors have metabolic particularities that, upon interaction within tumor microenvironment, would favor tumorigenesis. Analyzing both tumor cell metabolism and the metabolic outline of immune cells can offer innovative insights in new therapy targets and cancer therapeutical approaches. In addition to already approved immune- and targeted therapy in melanoma, approaching metabolic check-points could improve therapy efficacy and hinder resistance to therapy.

## Introduction

Metabolic requirements change when cells enter the proliferation process this is due to the fact that cell metabolism needs to sustain a different cellular stage. Accordingly, cancer cell needs to fulfill an increased biosynthesis rate because all cancer cells are characterized by a deregulated cell proliferation. Therefore, cancer cells have to direct available nutrients toward biosynthetic pathways maintaining ATP levels for a proper homeostasis. A drug targeting metabolic pathways could be common for many types of solid tumors (1). Nevertheless, normal cells when proliferate have the same metabolic needs to cancer cells, this raises the issue of targeting a metabolic pathways that can be common to normal and cancer cells, therefore finding the therapeutic window for an anticancer cell metabolism is still a matter of intense study.

Tumor consists of heterogeneous cellular populations and is subjected to various stimuli that would favor tumor development. These factors can be of neuroendocrine origin (2), UV irradiation (3), inflammatory conditions (4) or even, the still in debate, presence of Human papilloma viruses (HPV). HPV, a DNA virus, is strongly related to various non-melanoma cancers where 'high-risk' mucosal HPV consist mainly of 16, 18, 31, 33, and 35 types (5). The involvement of HPV in melanoma tumorigenesis has very few reports, in principle contradictory and mainly done on mucosal melanomas. So, there are groups that report the presence while other the lack of HPV infection. Thus, in mucosal melanomas HPV16 strain was found predominant while in skin melanoma HPV22 prevailed, the authors pointing out that HPV strains can be detected in some melanoma sub-groups (6). Similar results have shown that mucosal high-risk HPV genotypes are present in a quarter of precursor lesions acquired dysplastic melanocytic naevi and over a quarter in primary melanomas (7), or other HPV strains in melanomas (8). Other groups reported the lack of identifying the HPV in melanomas. Therefore, in primary malignant melanomas of non-sun exposed sites the lack of HPV detection was reported (9) as the same lack of HPV was reported in perineal melanomas (10).

The role of HPV infection in melanomagenesis is still a matter of debate and definitely needs further clinical and experimental investigations.

Due to this cellular heterogeneity the overall metabolic pattern of a solid tumor is *per se* heterogeneous starting from its genetic traits and ending with the variable microenvironment conditions where the tumor is developing. A series of drugs that target metabolism pathways has shown clear clinical benefits in trials (11). For example, L-asparaginase targeting aminoacid metabolism was already approved in acute lymphocytic leukemia; metformin alone or in combination for stage III-IV head and neck squamous cell cancer is in the clinical evaluation trials (12).

Intense preclinical studies performed on cell lines, primary tumor cells and *in vivo* models have shown that metabolic enzymes can be depicted as cancer therapy targets. Current concentrated studies efforts gather to understand tumor cell metabolism and all the factors that are conjoining to tumor's overall biological behavior. There is a common flow of events in tumorigenesis, and the most commonly accepted stages are the genetic events that activate signaling pathways for various deregulated cellular functions, including metabolic pathways. The fact that at molecular level deregulated cell's functions in tumorigenesis are linked with deregulated metabolic functions has open new therapeutic doors in cancer (13).

Another important point to be taken into account when investigating tumor cell metabolism is the fact that cancerous cells are in intimate contact with non-tumor cells, with various microenvironment structures and molecules (14) that will lead to the overall metabolic out-line of a tumor. Out of all non-tumor cells, immune cells that infiltrate the tumor are one of the most important cellular populations. In solid tumors, including melanoma and non-melanoma tumors, the tumor microenvironment (TME) is in the 5.7–7.0 pH range, therefore within the tumors, immune cells that infiltrate them will be subjected to this acidosis. Actually, innate and adaptive immune cells are regulated by acidic pH that is found generally in inflammation. Therefore, when immune cells infiltrate the tumor, they will be subjected to this acidic—inflammatory milieu. When immune cells are subjected to this acidic—inflammatory milieu they will trigger a series of events. Neutrophils will trigger anti-apoptosis events and differentiation process toward pro-angiogenic cellular patterns. Monocytes and macrophages will have their inflammasome activated inducing IL-1β synthesis. Conventional dendritic cells (cDC) will turn into a mature phenotype. All these cellular profiles indicate that innate immune cells recognize low pH as a danger-associated molecular pattern (DAMP). Adaptive immune cells will be as well-altered by low pH. T lymphocytes, with cytotoxic function will be repressed by low pH and IFN-γ production performed by T helper 1 (Th1) cells will be hindered. The mere raise in pH in the tumor microenvironment can reverse T lymphocyte anergy and enhance the antitumor immune response triggered by checkpoint inhibitors (15).

Therefore, in the attempt to review the metabolic profile of cutaneous melanoma, besides the actual metabolic profile of the tumor cell *per se*, immune cells that comprise the tumor microenvironment should be considered from the metabolic pathways point of view.

## Tumor Cell Metabolism in Melanoma

As already mentioned, the intense proliferation of a cancer cell can be sustained only partially by aerobic glycolysis, pathway that fuel macromolecules biosynthesis (16). In melanoma cells, in normal oxygen conditions, high glycolysis rate is encountered; 60–80% of the total glucose uptake is converted to lactate while in hypoxic conditions more than 90% will be converted in lactate (17). As generally recognized, oxygen accessibility is regulating metabolic outline of all cells. When the oxygen is low, hypoxia inducible factors (HIFs) will trigger molecular pathway that would adapt the cell to hypoxic stress (18). When HIF1 accumulates, the glycolytic rates increases, mitochondrial respiration decreases, while up-regulation of genes that enhance glucose uptake takes place. Transformed cells, like melanoma cells, have a constitutive HIF1 activation in both normoxia and hypoxia status (19). The increased glucose up-take in melanoma is sustained by the increased expression of transporter protein GLUT1 (SLC2A1) (20). The increased aerobic glycolysis augment lactate production mediated by lactate dehydrogenase A (LDHA) (21) one isoform converting lactate from pyruvate (22) while the gene encoding for LDHA is target for HIF1α. In the hypoxia status of tumor cells, glucose entering TCA cycle (tricarboxilic acid cycle) decreases and glutamine becomes the main carbon source (23).

Epigenetic metabolic studies in melanoma have shown that 5-hydroxymethylcytosine loss is linked with TET 5-methlycytosine hydroxylases family inhibition, this epigenetic deregulation is probably due to IDH2 (isocitrate dehydrogenase) down-regulation. Around 10% of melanoma tumor tissues have a mutant IDH1 or 2 (24). Melanoma cells harbor an amplified gene that encodes phosphoglycerate dehydrogenase (PHGDH), the first enzyme in the serine biosynthetic pathway and moreover one of the few acknowledged metabolic oncogenes (25). When PHGDH gene was silenced in cells, serine biosynthesis decreased, and tumor cells reduced their proliferation (26). If melanoma cell cultures are supplemented with exogenous serine, cellular proliferation continues to stay reduced in PHGDH silenced cells. Therefore, the mechanisms are linked with other pathways, such as cytosolic redox balance, amino acid transamination and probably many more still to be discovered. For example, serine leads to the formation of 5, 10-methylene-tetrahydrofolate, contributing to purine and pyrimidine biosynthesis, while glycine is essential for glutathione biosynthesis. Melanoma cells can redirect glucose toward serine and glycerol-3-phosphate pathways by enhancing phosphoenol pyruvate carboxykinase expression that is a cytosolic gluconeogenic enzyme. This process favors tumor cell proliferation, increasing glucose uptake and hence lactate production. If inducing in melanoma cells the silencing of phosphoenolpyruvate carboxykinase, tumorigenesis is reduced both in *in vitro* and *in vivo* models (27). Guanosine monophosphate reductase is involved in *de novo* purine biosynthesis and if the expression of guanosine monophosphate reductase is reduced, melanoma aggressiveness is enhanced. Decreasing intracellular GTP pools can limit melanoma cell's invasiveness as it was confirmed in invasive melanomas that guanosine monophosphate reductase is down-regulated (28).

Although new immune therapies have been approved for cutaneous melanoma (29, 30) the lack / poor clinical responses sustain the necessity to add new targets, such as altered metabolic enzymes / pathways that can aid or even can personalize therapy in melanoma.

In melanoma cells, as stated above, cytosolic serine pathway is upregulated. Inhibition of this metabolic pathway in other cancers (31) can be also extended to melanoma. Thus, if inhibiting serine biosynthetic pathway, oxidative stress can be induced in tumor cells. Higher ROS (reactive oxygen species) generation, reduces invasiveness because RHOA/GTP activity is decreased. Hypoxia drives glutamine pathways for fatty acid biosynthesis. Down-regulation of glycolysis upregulates oxidative phosphorylation to reinstate ATP levels needed for proliferation. Therefore, if BRAF (v-Raf murine sarcoma viral oncogene homolog B1) inhibitors can be combined with mitochondrial function inhibitors melanoma cell proliferation can be blocked at both levels. For example, introducing biguanides (metformin or phenformin) or glutaminase inhibitor BPTES the resistance to BRAF inhibitors will be clinically delayed (32).

Melanoma cells have a metabolic outline that gives the tumor cell advantages in an acidic—hypoxic milieu. However, a tumor complex architecture consists also of non-tumor cells, the main population being tumor infiltrating immune cells (TILs).

## Immune Cell Infiltrating Melanoma Tumors and Metabolic Traits

In developing melanomas, the immune cells that are the key anti-tumoral effectors would be summoned to the tumor site through a concert of molecules (33) and all these immune cells will have an important metabolic role (34). For example, in various cancers has been demonstrated that aldehyde dehydrogenase 7 family, member A1 (ALDH7A1) and lipase C, hepatic type (LIPC) expression is associated with negative and positive prognostic potency, respectively. In melanoma it was shown that the level of metabolic enzyme ALDH7A1 is correlated with low infiltrating immune cells. The metabolism of tumor cells impacts immune cells, microenvironmental inflammatory processes proving that “oncometabolism and immunometabolism” intersect (35).

When tumors have low immune cell infiltrates, more specifically low CD8+ CTLs the tumor can be resistant to therapy. In contrast tumors, with increased immune cell infiltrate, immunologically “hot” tumors, have an increased susceptibility to immune therapies (36). Immune cells and tumor cells within TME will race for resources, including glucose and amino acids, nutrients that sustain similar metabolic requirements (37). Moreover, due to their high metabolic rate, tumor cells will create a highly acidic, hypoxic, and rich in immunosuppressive metabolites TME, challenging the anti-tumoral action of immune cells. Therefore, immunotherapies that combine metabolic approaches that would favor immune cell infiltration, activate effector processes and enhance the life span of antitumor T cells would have increased clinical efficacy (38).

Main anti-tumoral effector immune cells, like cytotoxic T lymphocytes, NK cells, have metabolic outlines during their immune activity within TME.

### Cytotoxic T Cells Metabolism

Activation of T cells by T cell receptor (TCR) triggers also important metabolic changes. Un-stimulated T cells consume low levels of glucose and the metabolic energy is furnished by mitochondrial oxidative phosphorylation (OXPHOS). When T cells are activated and their functions demand there is a raise in the nutrient uptake, cells metabolism relying on aerobic glycolysis, glutaminolysis and lipid synthesis. Aerobic glycolysis enables T cells to use glucose to stimulate the pentose phosphate pathway (PPP). PPP produces NADPH, a molecule that is rate-limiting for the biosynthesis of amino acids, nucleic acids and fatty acids (37, 39, 40).

Tumor cells have an alike glycolytic metabolic pattern with activated T cells. TME will have a low concentration of nutrients, glucose, lipids and amino acids, thus lymphocytes will be suppressed by a lack of energetic molecules (41–43). T lymphocytes and other immune cells are inhibited by lactate or kynurenine, molecules secreted by tumor cells and additionally are subjected to low oxygen levels (41). In effector T cells, immune therapy with immune checkpoint blockade turns back the cell's metabolism to glycolysis (44, 45). PD-1 signals increase fatty acid oxidation (FAO), a pathway necessary for long-term survival of chronically activated T cells (44, 46).

Glycolytic metabolism is characteristic for active and efficient T lymphocyte, but in TME there are low levels of glucose (42, 43). Besides glucose deprivation, TILs have reduced enolase-1 activity, and this deficit can be overridden if downstream pyruvate can be supplied (47). Moreover, phosphoenolpyruvate (PEP), generated by enolase-1, has metabolically antitumor functions, it regulates the cytoplasmic Ca2+ and NFAT1 activation in T cells. When enhancing the expression of gluconeogenesis enzyme PEP carboxykinase 1, the intracellular PEP concentration leads to CD4+ or CD8+ T cells antitumor responses (43, 48). Glycolysis sustains in T cells cytokine production, cytolytic proteins and non-durable anti-tumor immune responses. Adoptive cell transfer (ACT) with memory T cells would sustain long-term antitumor responses (49). Therefore, in metabolic studies on memory immune cells approaches have focused on decreasing glycolysis in order to drive T cells toward the memory phenotype. Indeed, the glycolysis inhibitor 2-deoxyglucose differentiated T cells toward long-lived memory cells and enhanced the antitumor function. Therefore, memory T cells display low glycolytic metabolism and augmented OXPHOS characterizing a cell with medium cytotoxic function, self-renewal capacity and long lived cell (50). In melanoma, when ACT using melanoma-specific pmel T cells pretreated with the serine/threonine Pim kinases inhibitor (e.g., AZD1208) was applied an improved anti-tumoral response was obtained. In mouse melanoma model it was shown that when combining AZD1208 and PD-1 blockade, T cell ACT had an increased anti-tumoral effect (51).

Intra-tumoral Tregs are metabolically dependent on glycolytic pathway and recently it was demonstrated that TLR8 ligation would inhibit glucose uptake and glycolytic pathways (52). Furthermore, enhanced tumor glycolysis would lead to low therapeutic responses to ACT (53). Modulating *in vivo* glycolytic metabolism is a complex endeavor because targeting tumor cell metabolism would also impede TILs action.

One scope of an immune response is to develop memory cells, aim that is important in oncology where one would need the immune surveillance of metastasis. Memory T cells with anti-tumoral activity have metabolic particularities. When CD28 is co-stimulated in T cell priming, memory cell appears, and FAO pathways are enhanced, and morphological mitochondrial remodeling takes place. When CD28 co-stimulation does not take place, an active anti-tumoral response rapidly appears after ACT without generation of memory T cells for a durable response (54, 55). Inside TME, exhausted TILs are reported as being endowed with low or absent CD28 expression. In melanoma a costimulatory TNF receptor family member 4-1BB was found elevated on TILs with CD8+PD-1+Tim3+LAG3+ phenotype. In mouse melanoma 4-1BB co-stimulation increased mitochondrial function in CD8+ T cells and enhanced the efficacy of anti-check-point inhibitor (PD-1) therapy and T cell ACT (56).

Studies performed on other types of cancer (57, 58) show that targeting ROS has additional in antitumor effects. Targeting tumor cell mitochondrial metabolism in melanoma would enhance immunotherapies effect (59). Therefore, melanoma cell lines characterized by dysregulated oxidative metabolism triggered intratumoral hypoxia in mice while knockdown of mitochondrial complex 1 subunit Ndufs4 inverted the effect (59).

Briefly, anti-PD-1 therapy induces CD8+ T cell infiltration in melanoma tumors and tumor cell-intrinsic oxidative activity, while non-glycolytic tumor metabolism hinders anti-PD-1 efficacy.

**Figure 1:**
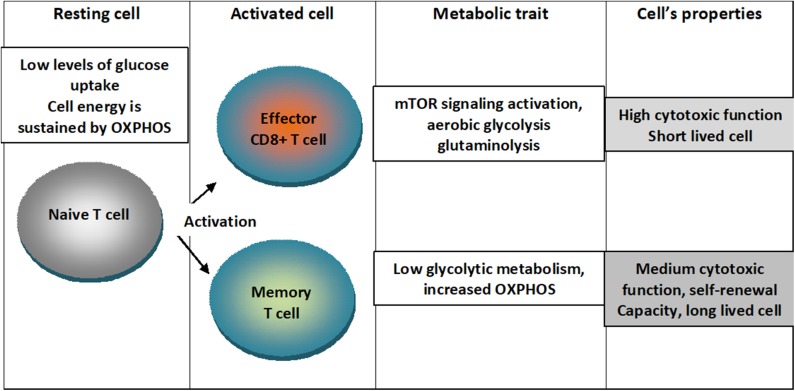
Metabolic traits of T cells upon differentiation in activated states. Naïve T cells are characterized by low levels of glucose uptake and cellular energetics is sustained by mitochondrial oxidative phosphorylation (OXPHOS). When cells are activated into effector CD8+ T lymphocytes, mTOR signaling is activated and cells switch to aerobic glycolysis characterizing a short lived cell with high cytotoxic function. When cells are activated into memory cell low glycolytic metabolism and augmented OXPHOS characterizing a cell with medium cytotoxic function, self-renewal capacity and long-lived cell.

An outline of the metabolic traits of T cells upon differentiation in activated states is depicted in Figure 1.

### NK Cells Metabolism

NK cells are involved in melanoma anti-tumoral response and their role is lately acknowledged as steadily increasing in melanoma (60). NK cells use glucose and upon activation they will up-regulate GLUT1 receptor increasing thus the fuel uptake. Activated NK would consequently increase the expression of transferrin receptor (CD71), receptors for large neutral amino acids (SLC7a5), glutamine (SLC1A5) and for free fatty acid (CD36). Glucose can be subjected in NK cells to two pathways, one leading to pyruvate and further to lactate that is accumulated in the cytosol; the second pathways is the conversion of pyruvate to acetyl-Co-A for uptake into the mitochondria. Acetyl-Co-A from the cytosol can enter lipid synthesis and many other acetylation reactions. The oxaloacetate (OAA) that is generated can further generate malate, re-entering the mitochondria, in the cycle Citrate Malate Shuttle (CMS). This cycle provides reducing equivalents for electron transport chain in OXPHOS (61). In the transition to effector cells NK nutrients uptake is increased (62) glycolysis and OXPHOS is increased in licensed NK cells in comparison to unlicensed NK that depend only on OXPHOS (63). Actually, licensed NK cells can be activated by MHC-I molecules to become functional (64).

### Dendritic Cells Metabolism

Dendritic cells (DC) are seminally involved in the anti-tumoral immune responses, but as in the other TILs, TME would hinder active DC function and dampen their anti-tumoral immune responses. DCs switch from immature (tolerogenic state) to the mature phenotype that would gain immunostimulatory activity upon sensing PAMPs and/or DAMPS *via* PRRs. The molecular flow of recognition is essential as further DCs activate through MHC-restriction effector lymphocytes CD8+ or CD4+ T cells. This activation takes place by the specific membrane complex of antigen-loaded MHC class I or II molecules, expressed on the DCs (65).

There are distinct DCs with specific metabolic requirements. Bone-marrow-derived DCs (BMDCs) in immature/resting state have fatty acid oxidation (FAO) as the basal pathway and OXPHOS in the mitochondrial electron transport chain (ETC) that supply the cells energetic necessities (66). In their resting state their functionality is low and would not activate T cells (67), but if PRR are activated, BMDCs rapidly switches to a pro-inflammatory state. This state is characterized by an increased expression of MHC molecules, cytokines and the costimulatory ligands (CD80 and CD86), so that antigen presentation would activate T cell mediated immune responses. Immediately upon activation, BMDCs would induce glycolysis pathways, this process being dependent on hexokinase II activation (68, 69). Stimulation *via* TLR2, TLR6, TLR9, Dectin-1 and Dectin-2, would reprogram as well DCs toward glycolytic pathways (70, 71). cDC in the early activation state will rely upon glycolytic metabolism from pre-stored glycogen. Glycolysis, TCA cycle and pentose phosphate pathway activation would ensure increased secretion of pro-inflammatory cytokines and T cell stimulatory molecules expression. After PRR activation, BMDCs would increase glucose transporter GLUT1 expression and increase glycolysis through the mTOR-HIF-1α/iNOS axis, inhibiting the electron transport chain. Upon these events, expression of MHC and co-stimulatory molecules would be decreased. In cDC, autocrine type-I IFN signaling would enhance HIF-1α-mediated glycolytic rewiring for further CD8+ T cell activation. There are still unknown mechanisms like the drivers for Mst1/2 activation and if cDCs use pyruvate for mitochondrial respiration (72).

**Figure 2:**
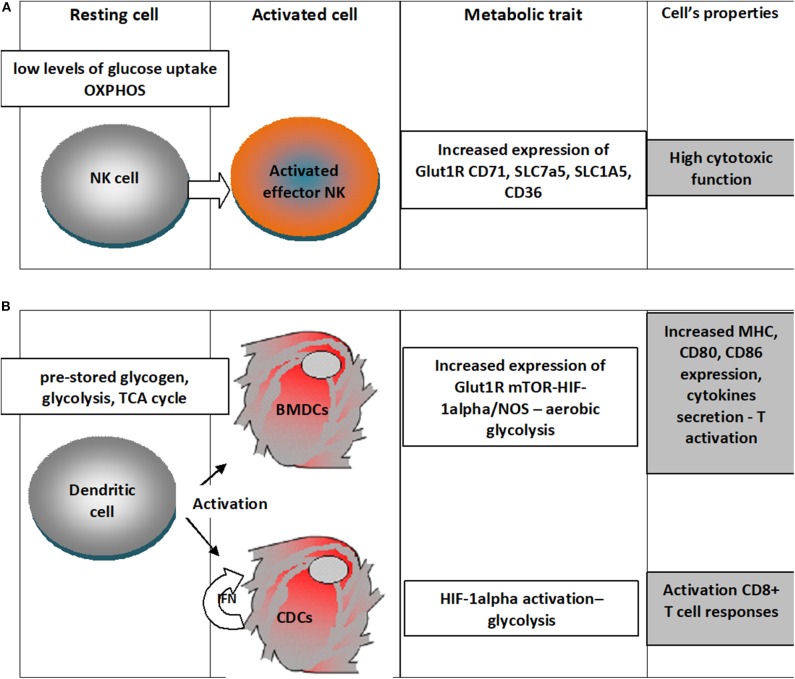
Metabolic traits of NK cells and dendritic cells upon differentiation in activated states. **(A)** NK cells in the resting state have low levels of glucose uptake and OXPHOS metabolism and upon activation there is an increased expression of Glut1R increasing thus the fuel uptake, would increase the expression of transferrin receptor (CD71), receptors for large neutral amino acids (SLC7a5), glutamine (SLC1A5) and for free fatty acid (CD36); all these changes sustain a high cytotoxic activity; **(B)** Dendritic cells in the resting state rely on pre-stored glycogen, use glycolysis, TCA cycle, when differentiating upon activation in Bone-marrow-derived DCs (BMDCs) increased expression of Glut1R mTOR-HIF-1alpha/NOS leads to aerobic glycolysis that will increase expression of MHC molecules, cytokines and the costimulatory ligands (CD80 and CD86); classical dendritic cells (cDC) would use the autocrine IFN activation to enhance HIF-1α-mediated glycolytic metabolism that would sustain CD8+ T cell activation.

An outline of the metabolic traits of NK and dendritic cells upon activation is depicted in Figure 2.

### Other Innate Immune Cells Infiltrating Tumors

Neutrophil effector functions are induced by PAMPs, DAMPs and several inflammatory cytokines. Their classical functions consist in phagocytosis and ROS generation. However, when the microenvironmental pH is lowered and hyperthermia installs anti-apoptotic processes are induced and other functions emerge, like a low phagocytosis, low ROS production, high expression of β2 integrins (CD11b/CD18), suppressive effects upon T cell responses and increased production of pro-angiogenic molecules (IL-8, VEGF, MMP-9) (73). Tumor acidosis would induce also in innate immune cells, like neutrophils, phenotypes that are similar with tumor-associated neutrophils (TANs), as described in various infiltrated tumors (74) and the existence of TANs is associated with a poor clinical prognosis in other cancers (75). TANs, influenced by the overall metabolic outline of the tumor would fav our pro-tumoral processes, metastasis, suppression of T cell effectors and neoangiogenesis (73, 74). Recent work of Holl et al. has shown that PDL1 expression on neutrophils in melanoma patients went up to almost 50% suggesting that PDL1+ neutrophils can have an immunosuppressive function (76).

Infiltrating immune cells sustain a major metabolic trait of melanoma being subjected to TME nutrients and oxygen restrictions that is similar to an inflammatory milieu.

## Therapy Related Metabolic Out Line in Melanoma

As stated in previous sections, the metabolic outline of cancer cells is represented by a metabolic reprogramming switching from oxidative phosphorylation to glycolysis. This switch favors cancer cell adaptation to a nutrient-poor microenvironment, favoring hence an aggressive cancer type. The possible therapeutic action on metabolic nodes was investigated for the already approved therapies in melanoma, namely upon targeted therapy (e.g., BRAF inhibitors) and on check-point inhibitors (anti—cytotoxic T-lymphocyte-associated protein-4—CTLA-4 and anti-programmed cell death-1 -PD-1 monoclonal antibodies) (see Table 1).

**Table 1:** Summarizes main metabolic points/pathways that can aid targeted/immune-therapy in melanoma and emphasizes that data obtained in *in vitro* and *in vivo* models prove that tumor's oxidative metabolism can be a future target to improve immunotherapeutic outcome for patients.

**Approved therapy**	**Targeted metabolic points**	**Drug**	**Effect**	**Reference**
Targeted therapy —BRAF inhibitor	HIFa/PDK3 pathway -	dichloroacetate, elesclomol	Glycolysis decrease, mitochondrial respiration enhanced through pyruvate dehydrogenase kinase-3 (PDK3) reduction	(79, 80)
	Ferroptosis	Agents inducing ferroptosis	Increase in targeted and immune therapy efficacy	(82–84)
	AMPK pathway	Phenformin	Inhibit mTOR signaling and induce apoptosis	(81)
Check-point inhibitors anti-PD-1	LDH pathway	Knockdown of LDH	Increase in tumor pH	(88–90)
	Mitochondrial pathway	UCP2 induction	Activation of DC and CD8+ T	(91)
	Tumor hypoxia	Metformin	Lowering tumor hypoxia	(94)
	Adenosine signaling	Antagonizing A2AR	Activation of T, NK and DC	(95)
Check-point inhibitors anti-CTLA4	Acetyl-CoA acetyltransferase-1 pathway	ACAT-1 inhibitor (avasimibe)	Abolishes CD8+ inhibition	(96)
	Essential amino acid pathway	IDO inhibitor	Abolishes tolerogenicity of immune cells in TME	(98–101)

### Targeted Therapy in Melanoma and Metabolic Pathways

BRAF targeted therapy resistance phenomenon appears early during therapy and influences patient's clinical outcome. The topic of therapy resistance in melanoma is still a matter of intense study. Several years ago, it was shown that there is an association between BRAF inhibitors and glycolitic pathways. A recent study has shown more specific in melanoma cell lines presenting BRAF mutation that their metabolic profiles influences the resistance to a BRAF inhibitor, Vemurafenib. Hence, clones of resistant cells are dependent on inflammatory lipid and EGFR signaling or have increased anaplerotic mitochondrial metabolism (77). In BRAF-mutant melanoma cells inhibition of BRAF induces concomitant glucose uptake reduction. Additionally, in melanoma cells various transcription factors regulate in parallel BRAF and glycolysis pathway. These transcription factors (e.g., HIF-1a, MYC, MLXIP) regulate glycolysis downstream of BRAFV600, and if concomitant inhibition is done, apoptosis is induced in cells that otherwise are resistant to BRAF inhibitor alone. This was the first proof-of-concept study that showed the therapeutic possibility to combine BRAF inhibitors with glycolysis inhibitor (78). The persistence of transcription factor HIF-1a at nuclear level in metastatic melanoma has opened other therapeutic possibility. If HIF-1a pathway can be blocked, glycolysis would decrease, and mitochondrial respiration could be enhanced through pyruvate dehydrogenase kinase-3 (PDK3) reduction. If PDK3 activity is inhibited (e.g., using dichloroacetate or specific siRNA) several molecular events tend to normalize. The study of Kluza et al. showed the preclinical validation that HIF-1/PDK3 bioenergetic route can be a new target for therapy in metastatic melanoma and can be used in patients that achieve BRAF-therapy resistance (79); further drugs aiming these targets (e.g., dichloroacetate, elesclomol) entered clinical trials (80).

There was reported a crosstalk between the AMPK (AMP-activated protein kinase) and BRAF signaling pathways. In cellular systems with BRAF-mutated melanoma cell lines when co-treated with phenformin (diabetes drug) and BRAF inhibitor (PLX4720) a decreased cell viability was registered. Moreover, phenformin treatment delayed resistance installment to BRAF inhibitor. It was shown that both drugs inhibit mTOR signaling and induce apoptosis. Furthermore, phenformin targets melanoma cells with slow cell cycling while PLX4720 targets increased cell cycling. Tumor regression was achieved in *in vivo* mouse models when using both drugs. Combining an AMPK activator with BRAF inhibitor can be a good therapeutical option in melanoma (81).

Another metabolic molecular event depicted in BRAF therapy resistance is the relation of aggressiveness and the specific sensitivity to iron-dependent oxidative stress, to ferroptosis, a specific cell death type. Therefore, drugs that induce ferroptosis can also increase targeted and immune therapy efficacy. Drugs that target dedifferentiation of melanoma cells in distinct stages through iron-dependent oxidative stress would better orient patient therapy (82). Using drugs that can induce ferroptosis is limited by bioavailable compounds. In GPX4 knockout melanoma cells it was demonstrated that increased ferroptosis sensitivity can be seen when combining ferroptosis-inducing drugs with BRAF inhibition (83, 84).

### Anti-Check-Point Inhibitors in Melanoma and Metabolic Pathways

Checkpoint inhibitors, anti-CTLA-4 and anti-PD-1 are approved in most of the European countries as therapies for melanoma, but still prediction markers for efficacy lack. A metabolic enzyme that is involved in the glycolytic activity of the tumor cells, LDH is the most important predictive marker for checkpoint inhibition efficacy. In fact, this enzyme reflects the acidity of TME, therefore in the metabolic therapeutical approaches this target should be followed (85). In a recently published report, it was shown that RNAi nanoparticle can neutralize tumor acidic environment and restore T cells action in the checkpoint blockade therapy. Within the study a knockdown of LDHA in tumor cells was induced, a reprogramming of pyruvate metabolism was induced, LDH was reduced and tumor pH increased. In an animal model, this proof-of-concept was verified. When tumor acidity was reduced, tumors had an increased infiltration of CD8+ T and NK cells, and reduced tumors growth. Moreover, PD-1 therapy was potentiated by this acidic TME neutralization (86). In another recent elegant study, it was shown that in patients tumors CTLs concentrate around peripheral blood vessels, especially in tumors with low TILs. *In vitro*, when subjected to low oxygen concentration and oxidative phosphorylation blockade it was shown that CTLs have reduced motility. This study highlights that hypoxia would limit effector immune cells migration favoring tumor cells to survive. Immunotherapy can be aided by normalizing pH and the intra-tumoral oxidative status (87).

As high LDHA expression is associated with poor prognosis (88) because acidic TME will block T cell cytokine production, hinder cytolytic function (88) and would induce NK cell apoptosis (89). In melanoma mouse model it was shown that raising tumor pH with sodium bicarbonate would slow the growth of Yumm1.1 melanoma and it enhanced anti-PD-1 or anti-CTLA-4 therapy efficacy (90).

In patients' tumors it was reported that metabolic deregulations are diverse in both degree and type. Therefore, some tumor cells have just deregulated oxidative or glycolytic metabolism, others have deregulated oxidative, but not glycolytic, metabolism that is associated to hypoxia. In single-cell melanoma models it was known that increased tumor oxygen consumption was associated with decreased T cell immune activity. In addition, melanoma cells without oxidative metabolism were responsive to anti-PD-1 therapy. Melanoma progression on PD-1 blockade is associated with an oxidative metabolism (59). The lack of T cells infiltrating the tumor resides also on the reduced expression of mitochondrial uncoupling protein 2 (UCP2) on tumor cells. It was shown that UCP2 reprograms TME inducing interferon regulatory factor 5 boosting type 1 DCs and CD8+ T cell action. Induction of UCP2 sensitizes melanomas to PD-1 therapy, alleviating primary therapy resistance (91).

In other cancers, it was shown that hypoxic non-vascularized areas that appear when tumor grow, are lacking infiltrating T cells (92) accordingly when reversing tumor hypoxia, the process of increasing T cell infiltration would aid immunotherapy. In 2018 it was shown that evofosfamide (TH-302), in an *in vivo* model restored normoxia, induced T cell infiltration in prostate tumors and enhanced the efficiency of PD-1/CTLA-4 checkpoint blockade (92). In a fibrosarcoma pulmonary mouse model, hyperoxia (60% O_2_) increased infiltration of antitumor CD8+T cells and increased CTLA-4/PD-1 efficacy (93). As in other types of cancers, this metabolic therapeutical approach can be applied as well in melanoma. Tumor hypoxia can be overridden by metformin potentiating PD-1 blockade in B16 melanoma mouse model (94). Adenosine signaling is another type of metabolic targeting that can modulate anti-check-point inhibitors therapy in melanoma. In hypoxic tumors A2A adenosine receptor (A2AR) is cross-linked it decreases the inflammatory process sustained by T lymphocytes, NK cells, DCs, and macrophages. Antagonizing A2AR a synergistic anti-tumoral effect with anti-PD-1 therapy can be induced (95).

It is known that cholesterol facilitates T cell receptor formation in CTLs, but an activation of CD8+ T cells increases acetyl-CoA acetyltransferase-1 (ACAT-1) and subsequently increases cholesterol esterification. In a melanoma animal model, an ACAT-1 inhibitor (avasimibe) was combined with immunotherapy and paclitaxel. The combination with avasimibe increased free cholesterol and abolished CD8+ T cells inhibition. The combination of avasimibe with immuno-therapy enhanced the antitumor action of CD8+ T cells (96).

Indoleamine 2,3-dioxygenase (IDO), is an enzyme that characterizes TME, this enzyme being produced by tumor cells, Tregs, stromal cells, and DCs. This enzyme turns the essential amino acid tryptophan (Trp) to kynurenine (Kyn). Low levels of Trp induces decreased mTORC1 signaling, but activation of GCN2 (stress kinase), drives cell cycle arrest and apoptosis in effector T cells (97). It was shown that high levels of IDO expression in the TME correlates with clinical outcome of the patients, increased resistance to PD-1 (98), increased resistance to CTLA-4 blockade (99) and to CD19-CAR-T cells (100). In mouse melanoma models an improved tumor control was obtained if anti-PD-L1 or anti-CTLA-4 was combined with IDO inhibitor INCB23843 (101).

### New Therapies Associated With Metabolic Pathways

A metabolic enzyme that gained tremendous attention in melanoma therapy is IDO1 as phase 1–2 clinical trials were developed to use IDO1 inhibitors in combination to anti-PD1 therapy. In spite of the high optimism, ECHO-301, phase 3 trial, was halted because IDO1- enzyme inhibitor (epacadostat) combined with anti-PD1 antibody (pembrolizumab) did not prove clinical benefits for patients. The failure of significant clinical results can come from several possible explanations. It is possible that in this trial insufficient drug exposure was done, hence the lack of effective inhibition. The preclinical data that sustain this trial show that DNA damaging drugs prove higher efficacy then IDO inhibitors (102). Therefore, studies are still needed to find dose/inhibitors types that target IDO and tryptophan 2, 3-dioxygenase in order to induce an improved blockade that can be associated to an improved clinical outcome.

Newly approved in melanoma, ACT can be inefficient in most tumors due to their molecular resistance mechanisms. Acknowledging that tumor glycolysis is associated with immune resistance in melanoma it was shown that over-expression of molecules from the glycolysis pathways impair T cell killing of tumor cells, while this anti-tumor action is enhanced by glycolysis inhibition in both *in vitro* and *in vivo* models. Genes that are involved in glycolysis pathways were found over-expressed in ACT-refractory melanoma patients. In enhanced glycolytic activity, melanoma cells have reduced levels of IRF1 and CXCL10, molecules that are involved in the immunostimulatory pathways and thus an association between tumor glycolysis and ACT efficacy was reported (53).

Virotherapy in melanoma is a newly opened field in clinical management of metastatic melanoma. Recently, a novel platform was reported for viruses that can induce an immune response targeted against tumor antigens. Hence, by attaching specific peptides on a Vaccinia virus and herpes simplex virus 1 (HSV-1) viral envelope a strong T cell-specific immune response was generated directed toward tumor antigens. Moreover, using HIV viral infection tactique, peptides can be enriched with Tat N-terminal peptide or conjugated with cholesterol. In mouse melanoma models it was demonstrated that by coating the viral envelope with peptides, the number of tumor-specific CD8+ T cells was enhanced (103). In the same immunotherapy domain, oncolytic viruses, can induce both tumor cell lysis and immune priming. The principle is clear, but adverse effects are still unclear. Recently it was shown that the oncolytic Vaccinia virus remodelates TME, that is dominated by effector T cells. In this milieu, leptin was identified as a potent metabolic reprogramming factor for an effective antitumor action. *In vitro* it was shown that leptin metabolically reprogrammed T lymphocytes, while melanoma tumor cells that expressed leptin were more efficiently attacked by effector cells. Using these experimental results authors engineered oncolytic viruses to induce leptin expression in melanoma cells and in mouse experimental model where completely regressed tumors were achieved and T memory populations was induced. This report from 2019 brings proofs that leptin provides metabolic support for anti-tumoral immunity using oncolytic viruses therapy (104). However, the molecular mechanisms are still to be clarified. In a recent mouse model using ob/ob and db/db strains the roles of leptin and resistin in the dacarbazine (DTIC) therapy in melanoma was reported. Leptin and resistin enhance proliferation of melanoma cell lines and hinder DTIC efficacy. Upon leptin and resistin treatment using A375 melanoma cell line, fatty acid synthase (FASN) and caveolin 1 (Cav-1), respectively, were found increased. Cell lines became more resistant to DTIC *via* upregulation of heat shock protein 90 (Hsp90) and P-glycoprotein (P-gp). These *in vitro* results pinpointed the adipokines involvement in melanoma progression, and other therapy resistance explanation (105).

New therapies that explore drugs modulating metabolic deregulations are still in their infancy and information is still to be gathered from preclinical and clinical studies.

## Conclusion

Tumor microenvironment is a network of complex cellular and molecular systems where cells will gain specific phenotypes / specific functions that would drive tumorigenesis. In skin cancers, tumor microenvironment is characterized by tumor infiltrating immune cells that sustain immune suppression, mainly regulatory T lymphocytes (106). Melanoma cellular heterogeneity is expressed on various levels, genetics (107), proteomics (108), transcriptomic (109), and metabolomic (110) and all these levels should be evaluated as closely interconnected. Besides several characteristics, melanoma cells display a metabolic reprogramming triggered by both genetic alterations and adaptation to a microenvironment that starts to lack nutrients and oxygen supply. Tumor cells have cytosolic and mitochondrial compartments that present clear metabolic adaptations to a demanding microenvironment and identifying deregulated glycolysis pathway could offer new therapy targets.

Moreover, the immune cells that infiltrate melanoma tumors have metabolic specificities that upon interaction within tumor microenvironment would favor tumorigenesis. In the last years implementing new immune therapies in melanoma has brought also an important clinical issue: resistance overcoming. Antibody-mediated blockade of immune checkpoint molecules implemented in melanoma therapy has shown that not all patients respond, that resistance is installed at one point and last, but not least immune-related adverse effects can be life threatening. Molecular mechanisms by which tumor cell becomes resistant are still to be uncovered (111, 112). Amidst these mechanisms, tumor and immune cell metabolism are gaining importance.

Analyzing both tumor cell metabolism and the metabolic outline of immune cells can offer innovative insights in new therapy targets and cancer therapeutical approaches. In addition to already approved immune- and targeted therapy in melanoma, approaching metabolic points could improve therapy efficacy and hinder resistance to therapy. In melanoma BRAF inhibition combined with drugs that target oxidative metabolism can lead to improvement in disease outcomes. In check-point inhibitors therapy targeting metabolic pathways can enhance anti-tumoral immune response and dampen therapy resistance.

Exploitation of tumor cell metabolism, tumor microenvironment and infiltrating immune cell metabolism could develop approaches that can aid current immunotherapies in melanoma.

## Author Contributions

MN has gathered data, written the paper, revised it and agreed to submission.

## Conflict of Interest

The author declares that the research was conducted in the absence of any commercial or financial relationships that could be construed as a potential conflict of interest.
